# Efficacy of Platelet-Rich Plasma on Symptom Reduction in Patellar Tendinopathy

**DOI:** 10.31486/toj.21.0031

**Published:** 2021

**Authors:** Katherine E. Fahy, Emily M. Miller, Yuka Kobayashi, Andrew W. Gottschalk

**Affiliations:** ^1^ Department of Family Medicine, Division of Sports Medicine, University of California, Los Angeles, Los Angeles, CA; ^2^ Department of Family and Sports Medicine, Oregon Health and Sciences University, Portland, OR; ^3^ Department of Orthopedics, Sports Medicine Institute, Ochsner Clinic Foundation, New Orleans, LA

## CASE PRESENTATION

A 19-year-old Division-1 collegiate soccer player presents to the training room with insidious onset right anterior knee pain. The pain started while she was doing rehabilitation following a right anterior cruciate ligament (ACL) reconstruction using a contralateral patellar tendon autograft. She has point tenderness over her right medial proximal patellar tendon, and she describes pain that worsens with repetitive loading of her knee during activities such as squats and running. She is diagnosed with patellar tendonitis via diagnostic ultrasound and starts physical therapy with a specific focus on eccentric and concentric loading of the quadriceps. She fails to improve following 3 months of compliant physical therapy and is frustrated by her continued pain. She asks about additional treatment options that would improve her pain.

## BACKGROUND

Subacute or chronic tendinopathy (or tendinosis) occurs in the setting of repetitive loading of a tendon that crosses a joint. While tendinopathy was initially thought to be an inflammatory process, research has demonstrated that tendon overuse activates protein kinases that induce apoptosis and disruption of the collagenous matrix in the tendon.^1^ These changes of the matrix can lead to alteration in how the tendon responds to loading and impact activities, resulting in pain that can negatively impact athletic performance.^1^

Patellar tendinopathy, or jumper's knee, occurs in the patellar tendon, which originates at the inferior pole of the patella and inserts on the tibial tubercle. This pathology typically presents as anterior knee pain at the inferior pole of the patella. The condition is a significant source of morbidity in both elite and recreational athletes, with one cross-sectional study suggesting that 33% of athletes are unable to return to their sport for more than 6 months.^2^

Conservative treatment of tendinopathy includes eccentric strengthening of the quadriceps, avoidance of aggravating activities, proprioceptive training, bracing, nitroglycerin patches, and heat therapy.^3^ Platelet-rich plasma (PRP) and autologous blood have also been proposed as treatments in tendinopathy.^4^ PRP has high concentrations of growth factors and bioactive molecules, including vascular endothelial growth factor, transforming growth factor beta 1, platelet-derived growth factor subunit A, platelet-derived growth factor subunit B, and insulin-like growth factor 1.^4^ The presence of these growth factors encourages cell and vascular growth, matrix formation, and differentiation that influence ligament, tendon, muscle, and bone healing.^4^

## REVIEW OF EVIDENCE

In a 2019 randomized controlled trial that included 3 treatment arms of 19 patients, all with symptoms for >6 months despite a prior trial of physical therapy and sonographic findings of tendinopathy, Scott et al compared injections of leukocyte-rich PRP (LR-PRP), leukocyte-poor PRP (LP-PRP), and saline. These injections were performed with ultrasound guidance, and after they received the injection, the athletes participated in rehabilitation that included concentric and eccentric loading under the supervision of a physical therapist for 6 weeks. By 12 weeks, 58% of participants had improved based on their Victorian Institute of Sport Assessment-Patella (VISA-P) score for pain and symptoms of patellar tendinopathy; however, no statistically significant difference was observed among treatment groups. The LR-PRP group had less improvement, although the difference was not statistically significant (35% of LR-PRP, 72% of LP-PRP, 71% of saline).^5^ One patient in the LP-PRP group reported pain at the injection site, but no other adverse events were reported.

Similarly, in 2014, Dragoo and colleagues compared ultrasound-guided dry needling alone with ultrasound-guided dry needling combined with PRP in 23 individuals with clinical evidence of patellar tendinopathy confirmed on magnetic resonance imaging (MRI) who were not responsive to conservative treatment. Both groups also participated in physical therapy twice weekly and eccentric strengthening at home as part of their rehabilitation. Patients who received PRP had a statistically significant (*P*=0.02) decrease in symptoms at 12 weeks (VISA-P score improved 25.4 ± 23.2 points, *P*=0.01 in the PRP group vs 5.2 ± 12.5 points, *P*=0.20 in the dry needling group). However, at 26 weeks, no statistically significant (*P*=0.66) difference between groups was observed (VISA-P score improved by 28.9 ± 25.2 points, *P*=0.01 in the PRP group vs 33.2 ± 14.0 points, *P*=0.001 in the dry needling group). No adverse events were reported in the PRP group.^6^

In contrast to the studies discussed above, Vetrano et al investigated the utility of multiple PRP injections compared to extracorporeal shock wave therapy in a sample of 43 participants with patellar tendinopathy. Two injections of PRP, 1 week apart, resulted in improved VISA-P and visual analog scale scores at the 6- and 12-month follow-ups, although no significant difference between groups was observed at the 2-month follow-up.^7^ To investigate the efficacy of multiple PRP injections, Charousset and colleagues conducted a prospective case series in which 28 athletes with refractory patellar tendinopathy of at least 4 months’ duration (mean 18 months with a range of 4-30 months) received 3 consecutive weekly LP-PRP injections from the same provider.^8^ Participants all had improved VISA-P scores at 2-year follow-up with no noted complications. At a mean time of 3 months, 75% of participants had returned to their previous sporting level. Seven athletes did not return to their previous sporting level: 3 returned at a lower level, 3 underwent surgery, and 1 switched sports for unrelated reasons. Corresponding with their return to sport, although no VISA-P scores were reported until 2 years after the PRP administration, MRI performed at 1 and 3 months post-PRP showed return to normal structural integrity in 57% of tendons and partial healing in 43%.^8^ Similar prospective case series have shown improved pain scores following single or multiple PRP injections.^9-13^

## TAKEAWAY

Few randomized trials have examined the effects of PRP on patellar tendinopathy. Those that exist are limited by small sample sizes, inconsistent treatment protocols, and different or lack of control arms. This inconsistency makes it difficult to determine with certainty the efficacy of PRP as a means of pain reduction in patellar tendinopathy.

While Dragoo et al and Scott et al showed no long-term benefits of PRP compared to dry needling and saline, both studies only included a single injection of PRP.^5,6^ In contrast, the study by Vetrano et al included 2 consecutive PRP injections and demonstrated a benefit from PRP.^7^ Further randomized controlled trials are needed to compare outcomes after single vs multiple PRP injections.

To complicate matters, multiple studies suggest that local anesthetics decrease the functionality of the platelets in PRP and reduce tenocyte proliferation.^14-16^ Dragoo et al injected bupivacaine and Scott et al injected lidocaine into the soft tissue surrounding the tendon.^5,6^ While the local anesthetic was not injected into the tendon or tendon sheath in either study, it is possible (if not likely) that the local anesthetic limited the potential therapeutic effect of the nearby PRP. Vetrano et al used multiple PRP injections but did not use local anesthesia.^7^

Despite a potential role for PRP in the treatment of patellar tendinopathy, further high-quality research with consistent and comparable study methods is needed to determine if PRP is a consistently effective treatment tool. Further research should focus on the optimal number of injections, use of local anesthetic, and postprocedure protocols (eg, a time frame for the transition from relative rest to formal physical therapy).

Adverse events related to PRP were rarely reported. The most common reported adverse event was pain at the injection site. Thus, PRP can be considered a safe treatment option. Physicians may consider using LP-PRP given that the limited available data suggest that LP-PRP is more beneficial than LR-PRP. Physicians can also consider multiple sequential injections of PRP, as this method may provide more benefit than a single injection. Based on the concern that local anesthetic may counter the therapeutic benefit of PRP, providers should attempt to avoid or at least minimize the use of anesthetics when performing these injections.

## CASE RESOLUTION

Using ultrasound guidance, the athlete underwent a patellar tendon LP-PRP injection without the use of local anesthetic. She was restricted from lower body activities for 2 weeks to allow tissue recovery. She then resumed her rehabilitation program with a focus on eccentric loading in addition to her post-ACL reconstruction exercise regimen. At 15-month follow-up, she was ready to play in the season opener for her collegiate team.

## Notes

*This article meets the Accreditation Council for Graduate Medical Education and the American Board of Medical Specialties Maintenance of Certification competencies for Patient Care and Medical Knowledge.*
